# Supercutaneous locking compression plate in the treatment of infected non-union and open fracture of the leg

**DOI:** 10.1007/s00402-021-04104-7

**Published:** 2021-08-04

**Authors:** Stein J. Janssen, Peter Kloen

**Affiliations:** 1 Department of Orthopaedic Surgery, Amsterdam Movement Sciences (AMS), Amsterdam University Medical Centre, University of Amsterdam, Meibergdreef 9, 1105 AZ Amsterdam, The Netherlands; 2 Department of Orthopaedic Surgery, Amsterdam Movement Sciences (AMS), Amsterdam University Medical Centre, Amsterdam, The Netherlands

**Keywords:** Supercutaneous plating, LCP, External fixator, External fixation, Frame, Nonunion, Pseudoartrosis

## Abstract

**Introduction:**

Salvage of infected tibia and fibula non-union and severe open fractures is challenging and often requires staged treatment. We describe all cases that underwent supercutaneous plating of the leg as external fixation technique and assessed union rate, time to union, rate of infection clearance, and patient-reported outcome measures.

**Methods:**

This is a retrospective cohort study from a single level 1 trauma center. We included 19 patients that underwent supercutaneous plating—locking compression plate applied as external fixator—of the leg. Indications were: infected non-union of a pilon, cruris, or ankle fracture (*n* = 13); post-traumatic fistula draining osteomyelitis of the tibia (*n* = 3); infected mal-reduced subacute cruris fracture (*n* = 1); acute open pilon fracture (*n* = 1); and acute open cruris fracture (*n* = 1). Outcome measures were: union, time to union, infection clearance, the 36-item Short Form (SF-36) physical component summary scale (PCS) and mental component summary scale (MCS), and NRS pain scores.

**Results:**

Union was achieved in 88% of the patients after a median of 279 days [interquartile range (IQR) 154–440]. Infection clearance was achieved in 94% of the patients. The PCS (median 51, IQR 46–56, *p* = 0.903) and MCS (median 57, IQR 50–60, *p* = 0.241) do not differ from normative population values. NRS Pain score at rest was 0 on average (IQR 0–1), 2 on average when walking (IQR 0–4), and 1 on average when climbing stairs (IQR 0–2).

**Conclusion:**

Supercutaneous plating is a simple and reliable technical trick to bridge and stabilize a nonunion or fracture site while clearing an infection and have soft-tissues heal before subsequent definitive (internal)fixation and/or cancellous bone grafting. Reasonable union and infection clearance rates are achieved, and good functional outcome can generally be expected.

**Level of evidence:**

Therapeutic level III.

## Introduction

Salvage of infected (distal) tibia and fibula nonunion and severe open fractures is challenging and often requires staged surgical treatment [1, 4, 6, 16]. Thorough debridement, removal or exchange of all implants in case of nonunion, and antibiotics are used to clear an infection, often followed by (definitive) reconstruction to achieve union in a 2nd stage [1, 4, 6, 16]. Mechanical stability at the nonunion/fracture site is often provided by an external fixator while the infection is being cleared and the soft-tissues heal [6, 16]. However, external frames can be heavy, bulky, and inconvenient to the patient. Our previously described technique of using a locking compression plate (LCP) as external fixator seems to be a valuable alternative to the conventional external frame [7, 9, 11, 17]. This “supercutaneous plating” technique has mostly been described in acute fractures in the past ten years [18]; only few papers describe its use in—infected—nonunion treatment. We therefore aimed to update our previous paper [17] on supercutaneous plating using a larger group of patients, with longer follow-up, and include patient-reported outcome measures. This helps understand the indications for, and usefulness of supercutaneous plating of the leg. We included all cases that underwent supercutaneous plating of the leg at our institution and describe union rate, time to union, rate of infection clearance, complications, and patient-reported outcome measures including physical function, mental function, and pain.

## Methods

### Study design

This retrospective cohort study included all consecutive patients that underwent supercutaneous plating of the tibia or fibula between November 2005 and December 2019 at our urban tertiary care referral center for complex orthopedic trauma. Patients were identified using our surgical case registry. We excluded 2 patients that underwent supercutaneous plating of other anatomical regions: one acute both bone open forearm fracture, and one infected ulna shaft nonunion [2]. All 19 remaining patients were included in this study. The indications for supercutaneous plating of the leg were: 13 (68%) patients had an infected nonunion of a pilon, cruris, or ankle fracture, 3 (16%) patients had a post-traumatic fistula draining osteomyelitis of the tibia (the fracture had healed), 1 (5.3%) patient had an infected mal-reduced subacute (26 days after injury) cruris fracture, 1 (5.3%) patient had an acute open pilon fracture, and 1 (5.3%) patient had an acute open cruris fracture (Table 1).

**Table 1:** Baseline characteristics of patients that underwent supercutaneous plating (*n* = 19)

	Median (interquartile range)
Age (in years)	53 (38–63)
Time between injury and supercutaneous plating (in days)	131 (40–884)

	*n* (%)
Male sex	14 (74)
Mechanism of injury	
High-energy blunt trauma	13 (68)
Low-energy blunt trauma	3 (16)
Crush injury	1 (5.3)
Unknown	2 (11)
Fracture type	
Closed	5 (26)
Open, grade 1	2 (11)
Open, grade 2	5 (26)
Open, grade 3 (not specified)	1 (5.3)
Open, grade 3A	3 (16)
Open, grade 3B	1 (5.3)
Unknown	2 (11)
Patients with other fractures at time of injury	9 (47)
Initial surgery at outside institution	12 (63)
Number of prior surgeries	
0	2 (11)
1	2 (11)
2	5 (26)
3	5 (26)
4 or more	5 (26)
Systemic comorbidities^a^	5 (26)
Indication for supercutaneous plating	
Nonunion cruris fracture	7 (36)
Nonunion pilon fracture	5 (26)
Nonunion ankle fracture	1 (5.3)
Cruris fracture infection, malreduction	1 (5.3)
Acute open cruris fracture	1 (5.3)
Acute open pilon fracture	1 (5.3)
Fistula draining osteomyelitis	3 (16)

^a^Rheumatoid arthritis, vascular claudication, hypertension, coronary heart disease, psoriasis, hypercholesterolemia, breast carcinoma

All patients were invited (in December 2020) to participate in this study by phone and completed a survey on paper sent by mail. Out of the 19 patients, 2 patients were deceased (6 and 11 years after surgery), 2 patients could not be reached, and one patient underwent below knee amputation because of persistent nonunion. Clinical data were collected for all 19 patients from the electronic medical record. Patient-reported outcome data were reported for the 14 patients that completed the survey (response rate: 82%, 14 out of 17 living patients). There was no standardized follow-up schedule due to the complexity of the cases and treatment; however, we followed patients until fracture union and infection clearance. Median overall follow-up was 3.7 years (IQR 1.2–8.7 years). The supercutaneous plating was used as the index procedure and date.

### Surgical technique

Preoperative imaging included standard anteroposterior and lateral radiographs of the leg, CT-scan to assess the nonunion/fracture site if unclear on radiographs, and whole leg standing radiographs were taken when clinical assessment suggested deviations of the normal mechanical axis. Infection parameters—serum erythrocyte sedimentation rate, C-reactive protein, and leucocytes—were tested at the preoperative consultation for nonunion cases. A plastic surgeon was consulted if a soft-tissue transfer (free or pedicled flap) was anticipated at any subsequent surgical stage. Duplex ultrasound or CT angiogram was performed —at the discretion of the consulted plastic surgeon—in case flap reconstruction was likely needed or vascular compromise was expected.

**Fig. 1:**
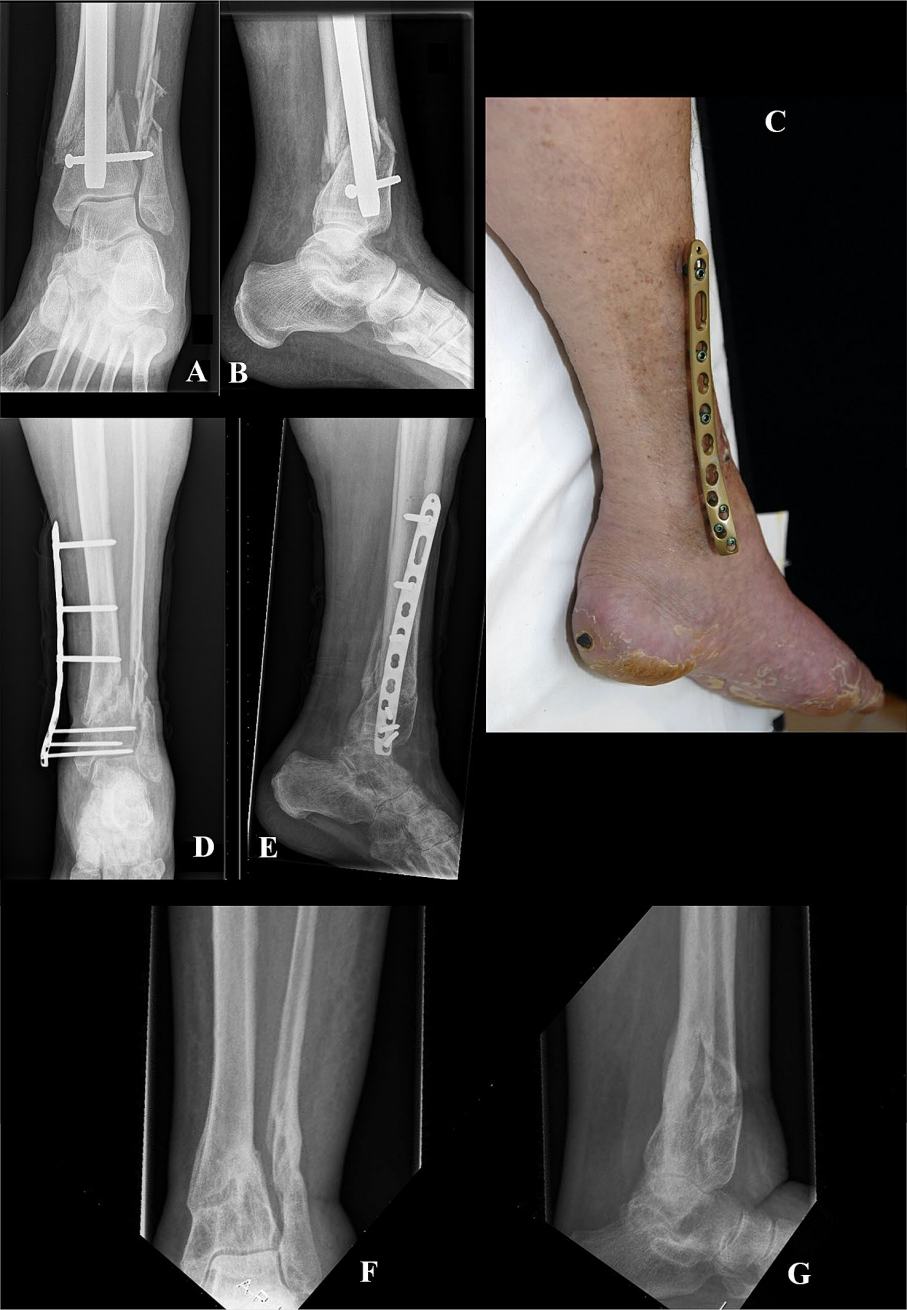
(Case 13 in Table 3): This patient was transferred to us from an outside institution 26 days after intramedullary nailing of a low-energy injury grade II open distal cruris fracture. The fracture was mal-reduced and infected (**A**, **B**). We removed the nail, performed a thorough debridement, took cultures and applied a metaphyseal locking compression plate 4.5/3.5 mm as external fixator (**C–E**). Cultures were positive for *Staphylococcus aureus* for which she was treated with clindamycin. The infection was cleared and union was achieved. The plate was removed after 154 days in the outpatient clinic, and no further surgery was required (**F**, **G**)

For the first surgical stage, the patient was under general or regional anesthesia and the involved leg was prepared in the standard sterile fashion. The nonunion site was approached through the previous surgical scar and five deep cultures were taken after which standard prophylactic antibiotics were given intravenously (2 g cefazolin). All implants (including broken screws) were removed and a thorough debridement performed, including opening the medullary canal if it was sealed off by sclerosis. If the reconstruction involved a Masquelet technique (*n* = 3, 16%), antibiotic cement (Copal G + V Bone cement containing 0.5 g gentamycin and 2 g vancomycin, Heraeus Medical, Heraeus Medical GmbH, Germany) was placed in the bony defect (defect sizes: 10 mm, 22 mm, and 62 mm). After mechanically aligning and optimized bony contact, we applied a titanium locking compression plate as an external fixator (Fig. 1). In general, we prefer the metaphyseal locking compression plate (3.5 mm and 4.5 mm screws) for nonunion of distal cruris or pilon fractures, and a broad 4.5 mm locking compression plate for nonunion of proximal cruris or shaft fractures (DePuy Synthes BV, Amersfoort, The Netherlands). The metaphyseal plate is specifically designed for the distal tibia with multiple points of fixation in an often short distal tibia segment. We prefer long plates to increase the number of screw options and aim for a 0.5 screw-to-hole ratio in the plate, meaning that about half of the plate-holes were filled with screws. Long (about 50–90 mm) titanium locking screws are placed via stab incisions, preferably 3–4 proximal, and 3–4 distal to the nonunion site with good bi-cortical purchase. The plate was placed close to the bone for optimal stability, yet allowed for some (± 1–2 cm) soft-tissue swelling. We checked plate and screw positioning using fluoroscopy intraoperatively. Nine cases (47%) had no bone defect; the defect size of the remaining 10 (53%) cases ranged from 10 to 62 mm (median 16 mm).

Patients were allowed to shower with the supercutaneous plate after 5 days when the surgical wound had healed. Patients were instructed to clean their percutaneous screw tracts daily with saline, and subsequently dress the wounds with gauzes. Patients were allowed to toe-touch or partially (up to 50%) weight bear, depending on the stability of the construct. All cultures were kept for 14 days. If two or more cultures were positive, a consultation with an infectious disease specialist was obtained to establish the appropriate antibiotic regimen. The next surgical stage(s) often involved definitive internal fixation, mostly combined with cancellous iliac crest graft; however, a wide variety of bone and soft-tissue reconstructions were needed.

### Explanatory variables and outcomes

The following baseline characteristics were collected from electronic medical records: age at time of supercutaneous plating, sex, mechanism of injury, fracture type [8], time from injury to supercutaneous plating, other fractures at time of injury, initial surgery at outside institution, number of prior surgeries of the affected leg, systemic comorbidities. The following surgical characteristics were collected from electronic medical records: number of screws in the supercutaneous plate (proximal and distal of fracture/nonunion site), screw–plate hole ratio (number of screws used divided by the number of screw options in the plate), time supercutaneous plate remained in situ, plate type used, plate location, bone defect size, location of plate removal (outpatient clinic versus in the operating room), intermediate screw removal including its reason, and number of subsequent surgeries of the affected leg. Defect size was measured on postoperative CT scans or radiographs. We also collected culture results and antibiotic regimens.

Outcome variables collected were: union based on bridging of at least 3 out of 4 cortices (yes/no), time to union, infection clearance (yes/no), and complications from medical records based on clinical assessment, laboratory values, and imaging. The patient-reported outcome measures completed via a survey included: the 36-item Short Form Health Survey (SF-36) patient-reported questionnaire assessing physical and mental health [5, 10, 15], numeric rating scale (NRS) pain scores (during rest, walking, running, and stair climbing), and use of pain medication. The SF-36 measures two distinct concepts: a physical component summary scale (PCS) and a mental component summary scale (MCS). Patients rate 36-items on 2-, 3-, 5-, and 6-point Likert scales. These were calculated and transformed to a general population average score of 50 with standard deviation 10 (range 0–100), with higher scores representing better physical or mental health [15].

### Statistical analysis

Variables are presented as frequencies with percentages for categorical variables and as median with (interquartile, IQR) range for continuous variables. Patients are presented case-by-case in a table. The PCS and MCS of the SF-36 are tested against the normative population average of 50 using the nonparametric signed-rank test. Statistical analyses were performed using Stata 16.0 (StataCorp LP, College Station, Texas). Continuous variables are presented as median with IQR because inspection of histograms suggested non-normality. There are missing values for: mechanism of injury (2 patients, 11%) and fracture type (2 patients, 11%).

## Results

### *Baseline and surgical characteristics (n* = *19)*

Median age was 53 years (IQR 38–63), the majority (68%) were men. Five (26%) patients had systemic comorbidities (Table 1). Most injuries were initially caused by high-energy trauma (68%), and open fractures were common (63%). Twelve out of the 19 (63%) patients had surgery at an outside institution before being referred to our center.

The median number of screws used in the supercutaneous plate was 7 (IQR 6–8), 3 proximal (IQR 3–4), and 4 distal (IQR 3–4). The median plate screw–hole ratio was 0.5 (0.38–0.5), indicating that half of the plate–holes were used on average (Table 2). The plate remained in situ for on average 100 days (IQR 56–142 days, range 2–477 days). The metaphyseal locking compression plate with 4.5 mm proximally and 3.5 mm screws distally was the most commonly used supercutaneous plate (63%) and plates were often placed on the medial side of the tibia (84%). Nine out of the 19 (47%) plates were removed without anesthesia in the outpatient clinic, which was well tolerated by the patient. The remainder were removed in the operating room during subsequent surgical procedures. Eleven screws were intermediately removed in six (31%) patients due to screw tract infection or local irritation. None of these screw tract complications leads to deep infection, sepsis, nor did removal compromise the stability of the construct. Almost all (95%) patients underwent one or more subsequent procedures after or with the supercutaneous plate in situ, including: further debridement (63%), cancellous iliac crest bone graft (42%), definitive internal plate or nail fixation (26%), arthrodesis of the ankle joint (10%), (osteo)cutaneous flap reconstruction (21%), split skin grafting (10), bone transport (5.3%), and below knee amputation (5.3%).

**Table 2:** Surgical characteristics of supercutaneous plating (*n* = 19)

	Median (interquartile range)
Number of screws	7 (6–8)
Number of screws proximal of fracture/nonunion site	3 (3–4)
Number of screws distal of fracture/nonunion site	4 (3–4)
Plate screw–hole ratio	0.5 (0.38–0.5)
Time supercutaneous plate remained in situ (in days)	100 (56–142)

	n (%)
Plate type	
Metaphyseal LCP 4.5/3.5	12 (63)
LCP 4.5	3 (16)
LCP 3.5	1 (5.3)
2 × LCP 4.5	1 (5.3)
2.7 LCP	1 (5.3)
Distal medial tibia LCP 3.5	1 (5.3)
Supercutaneous plate location	
Medial tibia	16 (84)
Medial and anterolateral tibia	1 (5.3)
Fibula	2 (11)
Removal of supercutaneous plate	
In the outpatient clinic without (local/general) anesthesia	9 (47)
In the operating room	10 (53)
Intermediate screw removal or screw exchange	
1 screw removed, local irritation, no antibiotics	2 (11)
2 screws removed, local irritation, no antibiotics	1 (5.3)
2 screws removed, due to screw tract infection	1 (5.3)
3 screws removed, due to screw tract infection	1 (5.3)
2 screws exchanged for better bone purchase	1 (5.3)
No screw removal or exchange	13 (68)
Number of subsequent surgeries	
0	1 (5.3)
1	6 (32)
2	7 (37)
3	2 (11)
4 or more	3 (16)

Out of the 19 cases, 3 did not grow any bacteria, 16 were culture positive of which 8 grew multiple species, and 8 only one species (Table 3).

**Table 3:** Individual patient data supercutaneous plate cases

Nr	Age > 50 years	Sex	Indication	Initial surgery at outside institution	Number of prior surgeries	Mechanism of injury	Fracture type	Time injury to plate (in days)	Time plate in situ (in days)	Plate screw-hole ratio	Number of screws	Plate location	Plate type	Bacterial species cultured	Number of subsequent surgeries	Union	Time to union (in days)	Infection clearance	NRS rest pain	NRS walking pain	PCS	MCS
1	Yes	Male	Nonunion pilon	No	1	High-energy	closed	28	94	0.5	7	Med. tibia	Distal med. tibia LCP3.5	*E. cloacae*	6	Yes	300	Yes	5	0	56	59
2	Yes	Female	Nonunion pilon	Yes	3	Low-energy	closed	166	134	4.0	4	Fibula	2.7 LCP	*E. coli*, P*. harei*	1	Yes	129	Yes	1	8	30	67
3	No	Female	osteomyelitis	Yes	3	High-energy	closed	884	104	0.4	6	Med. tibia	4.5 LCP	None	1	n/a	n/a	Yes	–	–	–	–
4	No	Male	Nonunion cruris	Yes	4	High-energy	open 3B	273	183	0.4	14	Med. AL. tibia	2 × 4.5 LCP	*S. aureus, P. aeruginosa*	2	Yes	183	Yes	–	–	–	–
5	No	Female	Nonunion cruris	Yes	7	High-energy	open 1	971	157	0.5	9	Med. tibia	Meta. LCP 4.5/3.5 plate	*S. aureus, E. coli, A. odontolyticus*	2	No	–	Yes	–	–	–	–
6	Yes	Female	acute open pilon	No	0	High-energy	open 3A	0	84	0.6	8	Med. tibia	Meta. LCP 4.5/3.5 plate	None	2	Yes	84	n/a	0	0	48	60
7	No	Male	Nonunion pilon	No	1	High-energy	closed	120	56	0.6	6	Med. tibia	Meta. LCP 4.5/3.5 plate	*S. aureus*	3	Yes	227	Yes	0	0	62	32
8	Yes	Male	Nonunion cruris	Yes	4	–	open 1	409	100	0.5	7	Med. tibia	4.5 LCP	*S. lugdunensis*	2	Yes	258	Yes	0	1	57	43
9	No	Male	Nonunion cruris	Yes	3	High-energy	open 2	1140	477	0.4	8	Med. tibia	Meta. LCP 4.5/3.5 plate	*S. aureus, P. aeruginosa*	4	Yes	477	Yes	0	1	51	55
10	Yes	Male	Nonunion cruris	No	3	High-energy	open 3A	131	56	0.5	11	Med. tibia	Meta. LCP 4.5/3.5 plate	*S. marcescens, S. aureus, E. faecalis*	2	Yes	440	Yes	0	2	55	56
11	Yes	Male	Nonunion cruris	No	2	Crush injury	open 2	113	101	0.5	8	Med. tibia	4.5 LCP	*S. aureus*	1	Yes	320	Yes	–	–	–	–
12	Yes	Male	osteomyelitis	Yes	2	–	–	8424	40	0.3	7	Med. tibia	Meta. LCP 4.5/3.5 plate	*S. pyogenes, S. aureus*	2	n/a	n/a	Yes	2	3	47	50
13	Yes	Female	infection cruris	Yes	2	Low-energy	open 2	26	154	0.6	6	Med. tibia	Meta. LCP 4.5/3.5 plate	*S. aureus*	0	Yes	154	Yes	–	–	–	–
14	No	Male	Nonunion cruris	No	2	High-energy	open 2	40	55	0.5	5	Med. tibia	Meta. LCP 4.5/3.5 plate	*E. faecium*	1	Yes	956	Yes	4	8	38	28
15	Yes	Male	osteomyelitis	Yes	4	High-energy	–	9140	66	0.3	6	Med. tibia	Meta. LCP 4.5/3.5 plate	*E. coli*	1	n/a	n/a	Yes	1	4	51	61
16	No	Male	acute open cruris	Yes	0	High-energy	open 3A	0	2	0.4	8	Med. tibia	Meta. LCP 4.5/3.5 plate	None	1	Yes	55	n/a	0	1	56	53
17	No	Male	Nonunion pilon	Yes	2	High-energy	open 2	106	27	0.6	9	Med. tibia	Meta. LCP 4.5/3.5 plate	*S. aureus, F. magna, S. hominis, S. mitis*	3	Yes	428	Yes	0	0	59	58
18	Yes	Male	Nonunion pilon	No	5	Low-energy	closed	52	142	0.3	6	Med. Tibia	Meta. LCP 4.5/3.5 plate	*E. faecium, E. cloacae, E. fecalis*	11	Yes	774	Quiescent	0	4	41	60
19	Yes	Male	Nonunion ankle	Yes	3	High-energy	open 3^a^	143	127	0.5	5	Fibula	3.5 LCP	*C. striatum*	2	No	–	Yes	1	2	46	61

– missing or unknown, *n/a* not applicable, *Med.* medial, *AL.* anterolateral, *Meta.* Metaphyseal

^a^Not specified

### *Clinical outcome measures (n* = *19)*

Union and union time were not applicable for 3 patients as these patients had osteomyelitis and an already healed fracture (Table 3). Of the remaining 16 patients, 14 had union (88%), 2 did not (12%). Time to union for the 14 patients that developed union was 279 days on average (IQR 154–440 days).

Infection clearance was not applicable in 2 acute fracture cases (Table 3). Of the remaining 17 patients, infection clearance was achieved in 16 patients (94%), in one patient, the infection is quiescent but not completely resolved (5.9%).

**Fig. 2:**
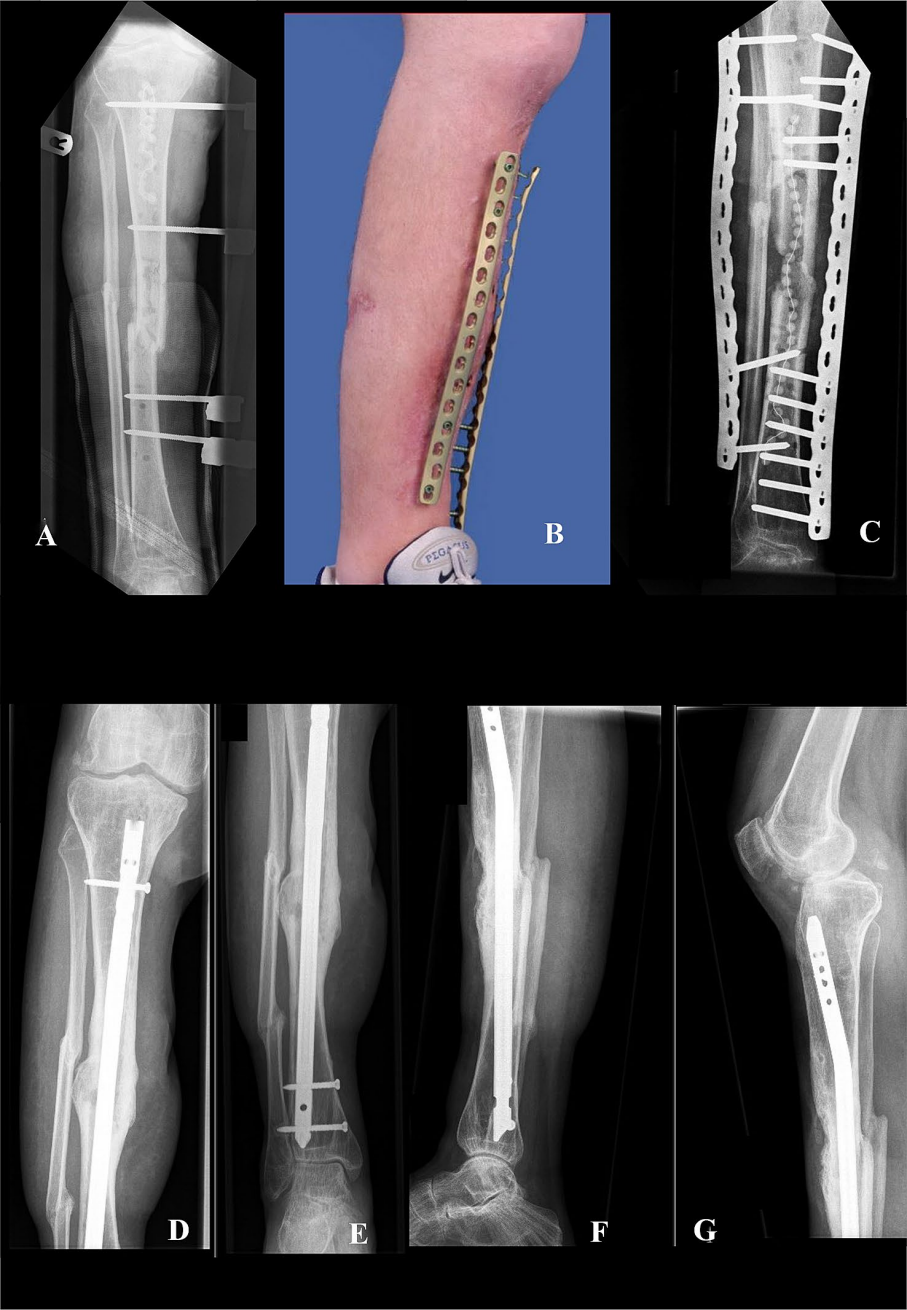
(Case 4 in Table 3): This patient was transferred to us from an outside institution a week after a motorbike accident in which he sustained a bilateral open cruris fractures (right leg grade 3B open fracture, left leg grade 2 open fracture) and an right knee dislocation. Both lower legs were initially stabilized at the outside institution with external fixators (**A**). A week after the injury we extended the right lower leg external fixator to the femur to achieve stability of the unstable knee, combined with wound debridement and a medial gastrocnemius flap to cover the soft-tissue defect of the right lower leg by the plastic surgeon. Cultures demonstrated *Staphylococcus aureus* and *Pseudomonas aeruginosa* for which he was treated with rifampicin, flucloxacillin, and ciprofloxacin. Several debridements, reaming of the intramedullary canal, and exchange of the external fixator ensued due to persistent infection of the right lower leg. We subsequently decided to remove the external fixator and place two broad 4.5 mm locking compression plates as external fixators to improve mobility and again thoroughly debrided the bone leaving gentamycin beats in the intramedullary canal (**B**, **C**). The infection cleared, and addition of cancellous iliac crest graft resulted in union. The supercutaneous plates were removed after 183 days in the outpatient clinic. Seven months later, he re-fractured his right leg during a mis-step for which he successfully underwent intramedullary nailing (**D**–**F**)

Complications were: distal cruris recurvatum of 16 degrees in one patient (no further surgery), post-traumatic ankle joint osteoarthritis in 2 patients which was moderately symptomatic (no further surgery), tibia re-fracture in one patient when making a misstep which was treated successfully by intramedullary nailing (Fig. 2), 10 degrees mal-rotation of the leg in one patient (no further surgery), below knee amputation because of persistent nonunion in one patient, and an infected fibular bone defect in one patient for which the distal fibula was removed when placing a hind-foot nail to fuse the severely affected post-traumatic ankle joint.

### *Patient-reported outcome measures (n* = *14)*

The physical component summary score (PCS median 51, IQR 46–56, range 30–62, *p* = 0.903) and mental component summary score (MCS median 57, IQR 50–60, range 28–67, *p* = 0.241) do not differ from normative population values (Table 3). Hence, physical function and mental health at latest follow-up seem comparable to general population scores.

NRS Pain score at rest was 0 on average (IQR 0–1, range 0–5), 2 on average when walking (IQR 0–4, range 0–8), 1 on average when climbing stairs (IQR 0–2, range 0–10). NRS pain on running was only scored by 5 patients with a median of 5 (range 0–10), the remaining patients indicated that they did not run, or were not able to run (Table 3).

Of the 14 patients, 3 patients (21%) used painkillers occasionally; acetaminophen by 2 patients, non-steroid anti-inflammatory drugs by 2 patients, tramadol by one patient.

The plate was well tolerated by patients, and it was possible to wear normal trousers over the affected leg and low-profile supercutaneous plate.

## Discussion

Salvage of infected (distal) tibia and fibula nonunion and severe open fractures is challenging and often requires staged treatment. We describe a cohort of 19 patients that had a locking plate fixation applied as an external fixator during their staged treatment. It is a simple and quick technique to stabilize the nonunion/fracture site while clearing the infection and have the soft-tissue heal—or reconstruct by a plastic surgeon—before subsequent definitive (internal) fixation. Our data show a reasonable union and infection clearance rate, and well tolerability of the plate with good patient-reported functional outcome on average. We therefore feel that this is a useful technical trick when treating infected nonunion of the leg.

This study has several limitations. First, it is a retrospective cohort study with inherent shortcomings. Selection bias played a role, as external fixators have also been used throughout the past 15 years at our institution. However, we generally use external fixators for relatively short time periods, and mainly in severe acute open fractures, whereas supercutaneous plating was more often used for longer time spans in the infected nonunion cases as presented. Second, generalizability of our results is unclear as this is a single surgeon case series. We see this as a major limitation; however, we feel that the technique is relatively simple and requires only limited resources. Third, the normative values of the SF-36 PCS and MCS are not validated in a large population; our findings did not differ from the calculated population norms, but it is unclear if these normative values are generalizable to the entire Dutch population.

Our 88% union, and 94% infection clearance rates are within ranges reported in the literature. A systematic review summarized the literature on surgical treatment of long-bone nonunion in 2007, 21 of the 34 included studies reported on tibial nonunion only (*n* = 640) and found union rates ranging from 66 to 100%, and infection clearance rates ranging from 0 to 100% among included studies [13]. The largest included cohort, published by Chan et al.[6], included 96 non-unions of the tibia treated by debridement, removal of implants, temporary filling of the defect with antibiotic-impregnated PMMA-cement beads, primary closure of the skin or flap transfer, and stabilization with an external fixator frame. At a later stage (2–10 weeks), the cement beads were removed when the infection had cleared and cancellous bone graft was packed into the defect; the external fixator frame was left in place until union. They achieved union in 99% of the patients, and infection clearance in 89%. A study by Toh and Jupiter described a series of 36 patients with 37 infected tibial non-unions—the majority also being open (59%) high-energy injuries initially—treated by radical serial debridements, removal of internal fixation and temporary external frame fixation between 1981 and 1991 [16]. After 61 months’ follow-up, they achieved union and infection clearance, both in 95% (35 of the 37) of the patients, one of whom eventually underwent below knee amputation (2.7%). Many patients had at least one pin tract complication. Hence, our results of using a supercutaneous plate as external fixator seems non-inferior—in terms of union and infection clearance rates—to external fixator frames traditionally used for staged tibial nonunion treatment reported in the literature. A systematic review by Bezstarosti et al. focuses on critical‐sized bone defects in the treatment of fracture‐related infection and includes 50 studies describing 1,530 patients (82% tibia defects) [3]. Techniques used to bridge/fill the defect were: cancellous bone grafting, induced membrane technique, vascularized grafts, and bone transport. They found an infection clearance rate of 83%, and an amputation rate of 3%. Cancellous and structural (vascularized) bone grafting, and the induced membrane technique can be combined with the supercutaneous plating technique presented in our paper. Bone transport, a reliable technique to bridge bone defects, is typically performed with an Ilizarov frame [12, 14]. A recently published retrospective cohort study presents a 95% maintained union rate in 102 patients treated with the Ilizarov frame for tibia nonunion [14].

We emphasize the importance of placing the titanium supercutaneous plate close to the bone to improve stability, yet with some clearance to allow for soft-tissue swelling. The plate should not compromise skin closure or future soft-tissue reconstruction. Plates can be contoured to accommodate leg anatomy and a free flap underneath. We aim to place about 3–4 screws above and below the non-union site, and fill about half of the screw holes with titanium bi-cortical locking screws. Alignment and bony contact should be optimized prior to placing the screws as the plate is more difficult to adjust then for example an external fixator. We feel that supercutaneous plating is a good alternative for conventional external fixation with a frame, especially in cases that require relatively long-term external stabilization (> 1 month). Hence, supercutaneous plating is a useful technique for treatment of infected non-union of the leg. The construct is lightweight and low profile without sharp ends or pins making it easier for patients to walk, exercise, and wear normal clothes. Potential problems and disadvantages are the fact that the plate and screws are not re-usable, and that the plate is harder to manipulate and adjust.

In conclusion, supercutaneous plating is a simple and reliable technical trick to bridge and stabilize a nonunion or fracture site while clearing an infection and have soft-tissues heal before subsequent definitive (internal) fixation and/or cancellous bone grafting. Reasonable union and infection clearance rates are achieved, and good functional outcome can generally be expected.
